# Osteoclast fusion and bone loss are restricted by interferon inducible guanylate binding proteins

**DOI:** 10.1038/s41467-020-20807-8

**Published:** 2021-01-21

**Authors:** David E. Place, R. K. Subbarao Malireddi, Jieun Kim, Peter Vogel, Masahiro Yamamoto, Thirumala-Devi Kanneganti

**Affiliations:** 1 Department of Immunology, St. Jude Children’s Research Hospital, Memphis, TN 38105 USA; 2 Center for In Vivo Imaging and Therapeutics, St. Jude Children’s Research Hospital, Memphis, TN 38105 USA; 3 Veterinary Pathology Core, St. Jude Children’s Research Hospital, Memphis, TN 38105 USA; 4 Department of Immunoparasitology, Osaka University, 3-1 Yamadaoka, Suita, Osaka 565-0871 Japan

**Keywords:** Cell signalling, Immunology, Osteoimmunology, Osteoclasts, Osteoarthritis

## Abstract

Chronic inflammation during many diseases is associated with bone loss. While interferons (IFNs) are often inhibitory to osteoclast formation, the complex role that IFN and interferon-stimulated genes (ISGs) play in osteoimmunology during inflammatory diseases is still poorly understood. We show that mice deficient in IFN signaling components including IFN alpha and beta receptor 1 (IFNAR1), interferon regulatory factor 1 (IRF1), IRF9, and STAT1 each have reduced bone density and increased osteoclastogenesis compared to wild type mice. The IFN-inducible guanylate-binding proteins (GBPs) on mouse chromosome 3 (GBP1, GBP2, GBP3, GBP5, GBP7) are required to negatively regulate age-associated bone loss and osteoclastogenesis. Mechanistically, GBP2 and GBP5 both negatively regulate in vitro osteoclast differentiation, and loss of GBP5, but not GBP2, results in greater age-associated bone loss in mice. Moreover, mice deficient in GBP5 or chromosome 3 GBPs have greater LPS-mediated inflammatory bone loss compared to wild type mice. Overall, we find that GBP5 contributes to restricting age-associated and inflammation-induced bone loss by negatively regulating osteoclastogenesis.

## Abstract (web summary)

The innate immune system and inflammation modulate bone homeostasis through complex regulation of bone remodelling cells including osteoblasts and osteoclasts. Here, the authors show that the type I interferon pathway and guanylate binding proteins functionally limit bone loss by inhibiting osteoclast functions.

## Introduction

Osteoclasts are myeloid-derived multinucleated cells involved in the resorption of bone^1^. Inflammation produced by chronic inflammatory disease or acute infection has been shown to drive the resorption of bone. The role of osteoclasts in this bone destruction is well established, and many inflammatory mediators have been implicated in driving osteoclast-mediated bone destruction, including IL-1, IL-6, IL-17, and TNF^2–4^. Autoimmune diseases, such as rheumatoid and psoriatic arthritis, and diseases associated with excessive inflammasome activation, such as cryopyrin-associated periodic syndromes, familial Mediterranean fever, or chronic recurrent multifocal osteomyelitis, are all associated with bone loss^5–7^. In addition, inflammation in patients with implanted devices, as commonly observed in total hip replacements, results in osteolysis and subsequent implant failure^8,9^. Infection-associated osteomyelitis, driven by infection with *Staphylococcus aureus* or *Porphyromonas gingivalis*, can also lead to localized bone loss^10,11^. Progressive loss of bone mineral density through aging results in osteoporosis, which increases the risk of fractures in the elderly, especially postmenopausal women^12,13^. Overly active osteoclasts contribute to many immune-driven inflammatory bone diseases, but they are also essential for normal formation of the bone marrow cavity, homeostatic bone remodeling and repair. Failure to generate osteoclasts through mutations in key regulators of their differentiation leads to a condition called osteopetrosis, which leads to densely formed bones that are prone to fracture. Given the importance of inflammation in driving destructive bone remodeling, it is important to understand how immune signaling pathways contribute to disease progression, and to identify potential new therapeutic targets for bone diseases.

The IFN pathway plays an inhibitory role in osteoclast generation^14–16^. Type I IFNs signal through IFNAR1/2 and the downstream proteins STAT1, STAT2, and IRF9, which together upregulate a number of IFN-stimulated genes (ISGs). Similarly, type II IFN (IFNγ) signals through the IFNGR and STAT1 homodimers, upregulating a distinct but also overlapping set of ISGs^17^. Upon stimulation with RANKL, a cytokine required for osteoclastogenesis, cells produce IFNβ, which acts as a negative feedback signal by inhibiting the expression of c-Fos, an essential transcription factor for osteoclast differentiation^14,18^. Mice lacking IFNAR1, IFNβ, IFNγ, or IFNGR1 have reduced bone density compared to wild type (WT) mice, highlighting the importance of IFN signaling in the negative feedback signaling and homeostasis of osteoclasts^14,15,19^. Similarly, IFNγ also suppresses osteoclastogenesis by suppressing TRAF6-mediated signaling downstream of RANK, the receptor for RANKL^16,20^. STAT1 and IRF1 critically regulate the expression of many ISGs, and osteoclasts deficient in either STAT1 or IRF1 undergo increased osteoclastogenesis in vitro^21,22^. Therapeutically, administration of IFNα to patients with bone loss secondary to mastocytosis, in combination with first-line treatment with bisphosphonates, increases bone density, and reduces the risk of fracture^23,24^. Other studies have shown that administration of IFNγ may prevent bone loss during rheumatoid arthritis and prevent bone loss in patients with postmenopausal osteoporosis^25^.

Better understanding of the mechanisms by which IFN and other ISGs regulate bone density is important in understanding how this immune pathway regulates bone homeostasis. Within the guanylate-binding protein (GBP) family of proteins, many are highly upregulated by IFN signaling^26,27^. These multifunctional proteins are large GTPases and many are poorly studied to date. Of these GBPs, GBP1, GBP2, and GBP5 are the best studied and are the only GBP family members to contain a C-terminal CaaX domain, which is cleaved and prenylated with farnesyl or geranylgeranyl lipid moieties, targeting them to membranes. Prenylation is common on small GTPases of the Ras, Rho, and Rab family proteins, including cdc42, Rac1, Rac2, Rap1, and Rab3d, which are each important in promoting osteoclastogenesis^28–35^. One of the front-line treatments for bone loss are nitrogen-containing bisphosphonates, which operate by inhibiting the prenylation of GTPases, resulting in osteoclast apoptosis^36,37^. GBPs have been shown to regulate actin dynamics in cells during bacterial infections and in IFNγ-treated cells, suggesting that they may regulate similar processes in osteoclasts^38–41^. Together with these observations and the critical role of IFN signaling in regulating osteoclastogenesis, we hypothesized that the IFN-inducible large GTPases (GBPs) regulate osteoclast differentiation and function.

## Results and discussion

### IFN signaling protects from bone loss and restricts osteoclastogenesis

**Fig. 1:**
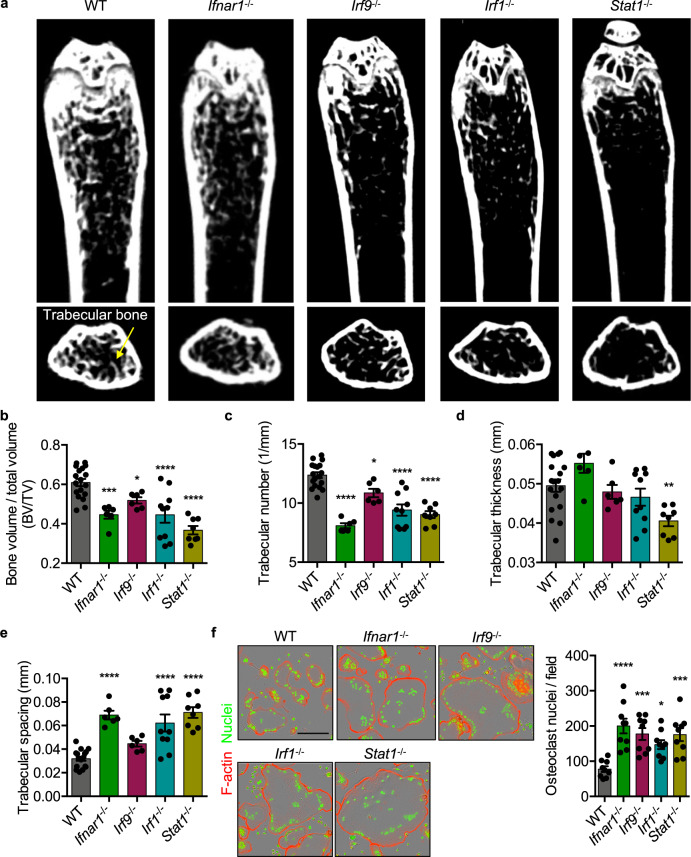
IFN signaling protects from bone loss and restricts osteoclastogenesis. Mouse femurs were scanned by micro-computed tomography (μCT) to compare bone morphometric parameters at 3 months of age. **a** 2D representative images of wild type (WT; *n* = 18), *Ifnar1*^−/−^ (*n* = 6), *Irf9*^−/−^ (*n* = 6), *Irf1*^−/−^ (*n* = 10), and *Stat1*^*−/−*^ (*n* = 8) femurs are displayed and quantitative measurements of **b** bone volume to total volume (BV/TV; WT vs *Ifnar1*^−/−^ *p* = 0.0003; WT vs *Irf9*^−/−^ *p* = 0.0238; WT vs *Irf1*^−/−^ *p* < 0.0001; WT vs *Stat1*^*−/−*^ *p* < 0.0001), **c** trabecular number (1/mm; WT vs *Ifnar1*^−/−^ *p* < 0.0001; WT vs *Irf9*^−/−^ *p* = 0.0156; WT vs *Irf1*^−/−^ *p* < 0.0001; WT vs *Stat1*^*−/−*^ *p* < 0.0001), **d** trabecular thickness (mm; WT vs *Stat1*^*−/−*^ *p* = 0.0038), and **e** trabecular spacing (mm; WT vs *Ifnar1*^−/−^ *p* < 0.0001; WT vs *Irf1*^−/−^ *p* < 0.0001; WT vs *Stat1*^*−/−*^ *p* < 0.0001) were compared. **f** Representative images (*n* = 9) of in vitro differentiated osteoclasts stained for F-actin (red) and nuclei (green) were collected by an automated IncuCyte S3 and osteoclast nuclei were quantified (WT vs *Ifnar1*^−/−^ *p* < 0.0001; WT vs *Irf9*^−/−^ *p* = 0.0003; WT vs *Irf1*^−/−^ *p* = 0.0138; WT vs *Stat1*^*−/−*^ *p* = 0.0004). Significance was determined (**b**–**f**) by one-way ANOVA followed by the Holm–Sidak multiple comparison test, *p < 0.05, **p < 0.01, ***p < 0.001,  ****p < 0.0001. Images and measurements are representative of pooled data containing at least six femurs per genotype (**a**−**e**) or from at least three independent experiments (**f**). Scale bar (black) indicates 200 μm (**f**). Data are presented as mean ± SEM (**b**–**f**).

Previous studies have shown that mice lacking key IFN signaling components have homeostatic changes in bone density, but have not been well compared in parallel^14,15,21,22,42,43^. We first reexamined, in age- and sex-matched mice, the role for type I IFN signaling molecules IFNAR1, IRF9, IRF1, and STAT1 in regulating bone density in femurs of 3-month-old mice by micro-computed tomography (μCT). Using μCT, we quantified bone morphometric data obtained from age- and sex-matched mouse femurs. Compared to WT mice, *Ifnar1*^−/−^, *Irf9*^−/−^, *Irf1*^−/−^, and *Stat1*^−/−^ mice had reduced bone density, as quantified by reduced bone volume to total volume (BV/TV) ratio, reduced trabecular number, and increased trabecular spacing, while only *Stat1*^−/−^ mice showed significantly reduced trabecular thickness (Fig. 1a–e). Our results in *Ifnar1*^−/−^ mice are consistent with published findings that *Ifnar1*^−/−^ mice have reduced bone density and increased osteoclastogenesis^14^. Earlier studies found that *Stat1*^−/−^ and *Irf1*^−/−^ mice have increased overall bone density, contrary to what would be expected from increased osteoclastogenesis in vitro^21,22^. In line with our data, the region distal to the growth plate of *Irf1*^−/−^ femurs has reduced BV/TV, while the trabecular spacing is increased^21^. These previous studies proposed that increased osteoblast activity in *Stat1*^−/−^ and *Irf1*^−/−^ mice was responsible for increased bone density. One proposed mechanism is that STAT1 limits nuclear translocation of the Runx2 transcription factor, limiting the proliferation of osteoblasts, and that STAT1 deficiency increases their replication^22^. Another proposed mechanism for increased bone density in *Stat1*^−/−^ mice is due to an increase in FGF18-dependent signaling in osteoblasts, and a decrease in the expression of *Cdkn1a* and *Fgfr3*, which limit the cell cycle^42^. In *Irf1*^−/−^ osteoblasts, the proposed mechanism for increased bone density is due to increased osteoblast proliferation and activity^21^. Because *Irf1*^−/−^ and *Stat1*^−/−^ mice were previously shown to have increased bone density, in contrast to our findings (Fig. 1)^21,22^, we utilized an additional independent genetic model and examined femurs from *Irf1*^fl/fl^*LysM*^Cre+^ mice, and also found an osteoclast-specific role for IRF1 in restricting bone loss (Supplementary Fig. 1). In the previous studies, *Irf1*^−/−^ bones were examined earlier (at 8 weeks) and bones were derived from *Stat1*^−/−^ mice on a different genetic background (129S6/SvEv-*Stat1*^*tm1Rds*^), suggesting these experimental differences may explain some of the observed differences with our data^21,22,42,44^. To further examine the role for these IFN regulatory proteins, osteoclasts were differentiated in vitro using recombinant M-CSF and RANKL. Consistent with the bone density reduction observed in *Ifnar1*^−/−^, *Irf9*^−/−^, *Irf1*^−/−^, and *Stat1*^−/−^ mice, in vitro osteoclasts derived from these mice exhibited enhanced osteoclastogenesis compared to WT (Fig. 1f). Consistent with our findings, patients with Mendelian susceptibility to mycobacterial diseases, defined by loss-of-function mutations in *IFNGR1* or *STAT1*, display reduced bone density and increased osteoclastogenesis^43,45,46^. Together, these data show that IFNAR1, IRF9, IRF1, and STAT1 each negatively regulate in vitro osteoclastogenesis and bone loss in mice.

### GBPs limit osteoclast fusion and bone loss

**Fig. 2:**
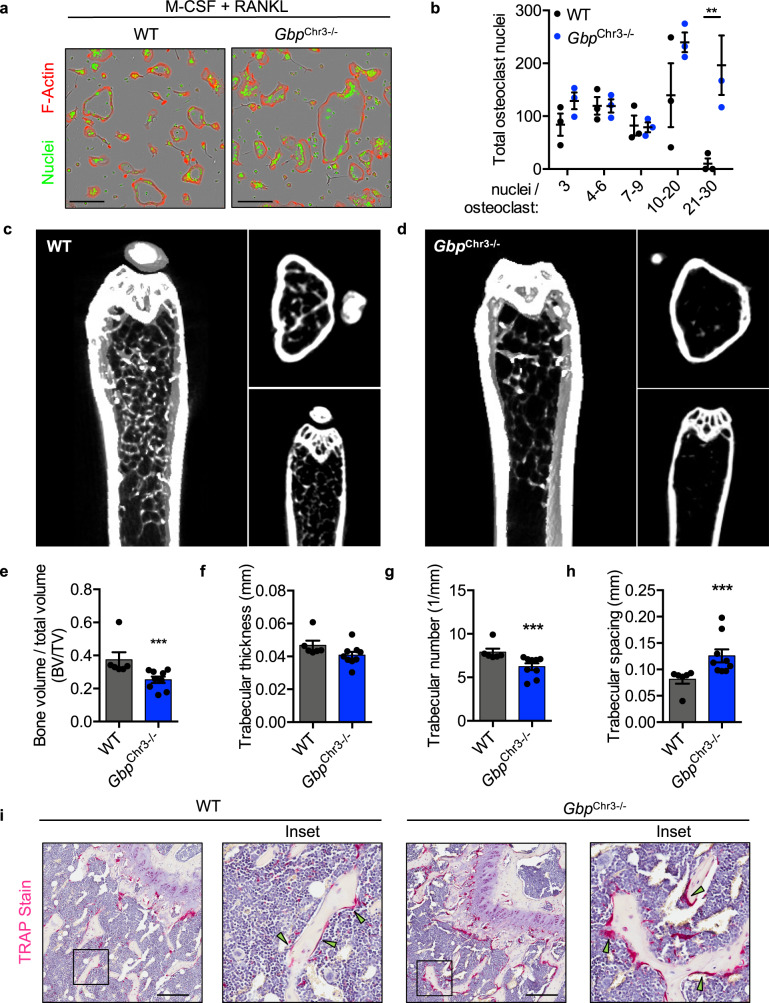
Guanylate-binding proteins limit osteoclast fusion and bone loss. **a** Representative images of in vitro osteoclasts from wild type (WT) or *Gbp*^Chr3−/−^ mice stained for F-actin (red) and nuclei (green). **b** The number of nuclei per osteoclast (>3 nuclei/osteoclast) was quantified by counting 12 images at 10× magnification per genotype (WT vs *Gbp*^Chr3−/−^ 21–30 nuclei osteoclasts *p* = 0.0013). **c**–**h** Representative 3D and 2D micro-computed tomography (μCT) sections were obtained from at least six femurs from 6-month-old **c**, **e**–**h** WT (*n* = 6) and **d**, **e**–**h** *Gbp*^Chr3−/−^ (*n* = 9) mice, and quantitative data of **e** bone volume to total volume (BV/TV; *p* = 0.0004), **f** trabecular thickness (mm), **g** trabecular number (1/mm; *p* = 0.0004), and **h** trabecular spacing (mm; *p* = 0.0004) were obtained. **i** Femur osteoclasts in 3-month-old mice were stained for tartrate-resistant acid phosphatase (TRAP) activity (magenta; green arrow), counterstained with hematoxylin, and imaged at the indicated magnification. Significance was determined by **b** two-way ANOVA with Sidak’s multiple comparison test or **e**−**h** two-tailed Student’s *t* test, **p < 0.01, ***p < 0.001. Scale bars (black) indicate 200 μm (**a**, **i**). Data are presented as mean ± SEM (**b**, **e**–**h**). M-CSF macrophage colony-stimulating factor, RANKL receptor activator of nuclear factor-κΒ ligand.

IFNs potently inhibit osteoclastogenesis and lead to the expression of hundreds of ISGs following stimulation^14^. The role of the interferon-inducible GBPs in osteoclasts, to our knowledge, has not been explored. Given that GBPs are highly upregulated following IFN stimulation^26,47^, we examined whether osteoclasts deficient in five GBPs on mouse chromosome 3 (GBP1, GBP2, GBP3, GBP5, and GBP7, indicated as *Gbp*^Chr3−/−^) would form larger osteoclasts similar to those differentiated from *Irf9*^−/−^, *Irf1*^−/−^, and *Stat1*^−/−^ mice. Consistent with the increased osteoclast fusion observed in IFN signaling-deficient cells (Fig. 1), osteoclasts derived from *Gbp*^Chr3−/−^ mice fused to a greater extent than WT cells did (Fig. 2a). The extent of multinucleation in osteoclasts which contained at least three nuclei was quantified. Osteoclasts differentiated from *Gbp*^Chr3−/−^ mice contained more nuclei than WT osteoclasts, suggesting that expression of GBPs negatively regulates osteoclast fusion (Fig. 2b). Live cell imaging of WT and *Gbp*^Chr3−/−^ cells further showed that GBPs negatively regulate osteoclast fusion (Supplementary Movies 1 and 2). To determine whether GBPs contribute to the negative regulation of osteoclast activity in vivo, femurs were collected from 6-month-old mice, and bone morphometric data were collected by μCT. Consistent with increased in vitro osteoclastogenesis and similar to the bone loss observed in IFN signaling-deficient mice (Fig. 1), femurs from *Gbp*^Chr3−/−^ mice had reduced bone density (BV/TV) and trabecular number, and increased trabecular spacing compared with WT mice with a trend toward reduced trabecular thickness (Fig. 2c–h). To determine whether osteoclasts were enhanced in *Gbp*^Chr3−/−^ femurs, bones were decalcified, sectioned and stained for tartrate-resistant acid phosphatase (TRAP), a marker of mature osteoclasts^48^. Consistent with our μCT findings, bones of *Gbp*^Chr3−/−^ mice showed more trabeculae-associated osteoclasts compared with WT mice (Fig. 2i). Together, these data reveal that GBPs can negatively regulate osteoclastogenesis under homeostatic aging-dependent bone turnover. Because GBPs have been found to regulate the polymerization of F-actin during infections and Arp2/3-mediated cytoskeletal rearrangements are required for driving osteoclast fusion, the loss of GBPs may mechanistically increase fusion through altered cytoskeletal remodeling^39–41,49–51^. Consequently, loss of GBPs increased Arp2/3-dependent actin polymerization during infection with *Burkholderia thailandensis*, resulting in significantly increased macrophage cell–cell fusion, consistent with the observed increased osteoclast fusion phenotype, suggesting GBPs regulate actin dynamics across cell types^52^. How GBPs specifically inhibit the polymerization of F-actin via the Arp2/3 complex is an outstanding question in the GBP field^27,53,54^. The mechanisms behind osteoclast fusion are similarly poorly understood, and these data suggest GBP-mediated regulation of cell fusion may be important for inflammatory bone remodeling.

### GBP5 but not GBP2 restricts bone loss in mice

**Fig. 3:**
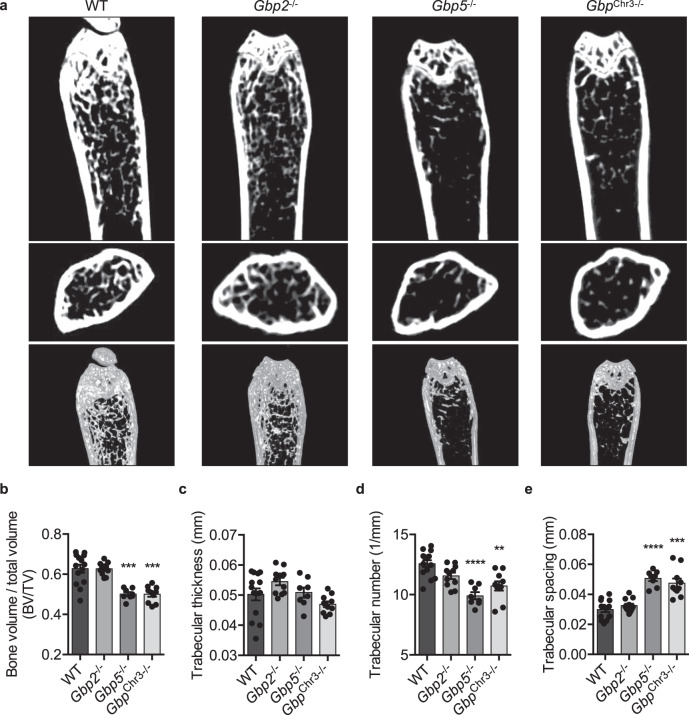
Guanylate-binding protein 5 (GBP5) but not GBP2 restricts bone loss in mice. Mouse femurs were scanned by micro-computed tomography (μCT) to compare bone morphometric parameters at 3 months of age. **a**–**e** Representative 2D and 3D images of wild type (WT; *n* = 14), *Gbp2*^−/−^ (*n* = 11), *Gbp5*^−/−^ (*n* = 8), or *Gbp*^Chr3−/−^ (*n* = 10) femurs collected following μCT scans and quantitative **b** bone volume to total volume (BV/TV; WT vs *Gbp5*^−/−^ *p* = 0.0006; WT vs *Gbp*^Chr3−/−^ *p* = 0.0008), **c** trabecular thickness (mm), **d** trabecular number (1/mm; WT vs *Gbp5*^−/−^ *p* < 0.0001; WT vs *Gbp*^Chr3−/−^ *p* = 0.0049), and **e** trabecular spacing (mm; WT vs *Gbp5*^−/−^ *p* < 0.0001; WT vs *Gbp*^Chr3−/−^ *p* = 0.0003) measurements were collected from at least six femurs. Significance was determined by **b**−**e** one-way ANOVA followed by the Holm–Sidak multiple comparison test, **p < 0.01, ***p < 0.001, ****p < 0.0001. Data are presented as mean ± SEM (**b**–**e**).

Because *Gbp*^Chr3−/−^ mice lack five GBPs (GBP1, GBP2, GBP3, GBP5, and GBP7), we sought to determine which GBPs within this locus contribute to the regulation of osteoclastogenesis and bone loss. Among the best studied murine GBPs are GBP2 and GBP5, and previous studies found that both were required for cell-autonomous immunity against the intracellular bacterium *Francisella novicida*^55,56^. Few studies have shown distinct functions for GBP2 and GBP5, though the proteins are thought to differ in their localization, suggesting that each may have unique functions^57^. While their individual functions are poorly understood, GBP2 has been shown to play a larger role in recognition of *Escherichia coli* outer membrane vesicles, while GBP5 has been shown to play a dominant role in mediating *Brucella abortus* LPS-dependent activation of caspase-11 (refs. ^58,59^). Both GBP2 and GBP5 are prenylated to facilitate membrane localization, similar to other small GTPases that regulate osteoclastogenesis^29,60,61^. To determine the contribution of each of these GBPs to bone density, femurs from the respective gene deficient mice were collected, and bone morphometric data were collected by μCT. Both *Gbp5*^−/−^ and *Gbp*^Chr3−/−^ mice, but not *Gbp2*^−/−^ mice had reduced bone density as measured by BV/TV, trabecular number, and trabecular spacing (Fig. 3a–e). In vitro osteoclasts derived from *Gbp2*^−/−^, *Gbp5*^−/−^, or *Gbp*^Chr3−/−^ mice all have increased osteoclastogenesis compared with WT mice, suggesting that in vivo, GBP2 is not necessary for normal bone maintenance (Fig. 3a–e and Supplementary Fig. 2a, b). Mechanistically, increased cell–cell fusion in GBP-deficient osteoclasts is likely due to changes in actin cytoskeleton dynamics, as expression of required osteoclast fusion mediators DC-STAMP and ATP6V0D2 are similar to WT osteoclasts (Supplementary Fig. 2c). How GBPs regulate actin cytoskeleton dynamics is an outstanding question in the field^27,54,62^. While it is surprising that GBP5 alone regulated bone density in mice, it is possible that the loss of GBP2 causes a compensatory response that counteracts any changes in osteoclastogenesis in vivo. Indeed, previous work has shown that human GBP1, which most closely phenocopies mouse GBP2, is upregulated in mesenchymal stromal cells which give rise to osteoblasts and increased expression is associated with osteoporosis^63,64^. Together, these data show a distinct role for GBP5 in negatively regulating bone loss in mice during homeostasis.

### GBPs limit acute inflammatory bone loss

**Fig. 4:**
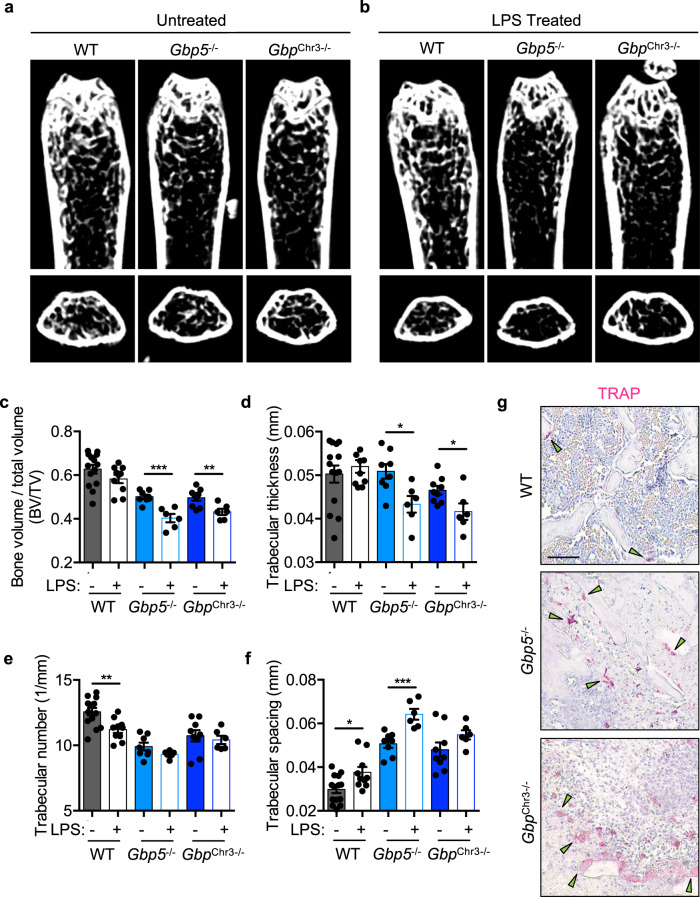
Guanylate-binding proteins limit acute inflammatory bone loss. Acute bone loss was measured by comparing untreated 3-month-old femurs and femurs from mice treated with two doses of lipopolysaccharide (LPS) for a total of 8 days. Representative 2D micro-computed tomography (μCT) images were collected from **a**, **c**–**f** untreated control wild type (WT; *n* = 14), *Gbp5*^−/−^ (*n* = 9), or *Gbp*^Chr3−/−^ (*n* = 8), and **b**, **c**–**f** LPS-treated WT (*n* = 10), *Gbp5*^−/−^ (*n* = 6), and *Gbp*^Chr3−/−^ (*n* = 6) mice. Quantitative measurement of **c** bone volume to total volume (BV/TV) (*Gbp5*^−/−^ *p* = 0.0004; *Gbp*^Chr3−/−^ *p* = 0.0071), **d** trabecular thickness (mm; *Gbp5*^−/−^ *p* = 0.0116; *Gbp*^Chr3−/−^ *p* = 0.0253), **e** trabecular number (1/mm; WT *p* = 0.0028), and **f** trabecular spacing (mm; WT *p* = 0.0127; *Gbp5*^−/−^ *p* = 0.0008) were compared between untreated control and LPS-treated mice. **g** LPS-treated mouse femur tartrate-resistant acid phosphatase (TRAP)-positive osteoclasts were stained (green arrow). Statistical significance was determined by **c**−**f** two-tailed Student’s *t* test, *p < 0.05, **p < 0.01, ***p < 0.001. Untreated control data from multiple independent pooled scans were compared to a single LPS injection experiment (*n* = 6–10 femurs). Scale bar (black) indicates 200 μm **g**. Data are presented as mean ± SEM (**c**–**f**).

To further examine the role of GBPs in regulating bone turnover in mice, we utilized an acute bone loss model where LPS injection induces rapid bone loss and osteoclastogenesis^2,14,65,66^. Mice were injected intraperitoneally with 5 mg/kg LPS at days 0 and 4, and bones were collected at day 8, as previously described^66,67^. Compared with untreated control mice, LPS injection resulted in significantly greater bone loss in *Gbp5*^−/−^ and *Gbp*^Chr3−/−^ mice (Fig. 4a–f). To determine the effect of LPS on osteoclastogenesis in vivo in GBP-deficient mice, bone sections were stained for osteoclasts using a TRAP stain. Consistent with the observed increase in bone loss in *Gbp5*^−/−^ and *Gbp*^Chr3−/−^ mice compared with WT mice, osteoclasts associated with trabecular bone were increased in both *Gbp5*^−/−^ and *Gbp*^Chr3−/−^ femurs (Fig. 4g). Together these data establish a critical role for GBP5 in negatively regulating inflammatory bone loss. Future studies examining GBP expression during inflammatory bone loss may reveal important regulatory networks that were previously not appreciated. Because GBPs contribute to both age-related and acute inflammation-related bone loss, they likely function during chronic low-grade inflammation driven by microbiota and during acute infection-induced osteomyelitis. This regulation of inflammatory bone loss may lead to new strategies for treating inflammation-driven bone diseases.

## Methods

### Mice

WT (C57BL/6J), *Gbp2*^−/−^ (*Gbp2*^*tm1b(KOMP)Wtsi*^*)*^55^*, Gbp5*^−/−^^55,68^*, Gbp*^Chr3−/−^ (MGI:5438974*)*^69^*, Ifnar1*^−/−^ (*Ifnar1*^*tm1Agt*^*)*^70^*, Irf9*^−/−^ (*Irf9*^*tm1Ttg*^*)*^71^*, Irf1*^−/−^ (*Irf1*^*tm1Mak*^)^72^, and *Stat1*^−/−^ (*Stat1*^*tm1Dlv*^)^73^ mice were backcrossed to the B6 background. Myeloid-specific *Irf1* knockout mice (*Irf1*^fl/fl^*LysM*^Cre+^) were generated by crossing *Irf1*^fl/fl^ mice (B6Dnk;B6Brd;B6N-*Tyr*^c-Brd^*Irf1*^tm1a(EUCOMM)Wtsi^/WtsiOulu; Infrafrontier, EM:05519) and *LysM*^Cre+^ mice (*B6.129P2-Lyz2*^*tm1(cre)Ifo*^/J*)*, and littermate controls were used. Male mice were used in this study at indicated ages (3 or 6 months) or 6–8 weeks old (for generation of in vitro osteoclasts). Mice were bred at St. Jude Children’s Research Hospital, and studies were conducted under protocols approved by St. Jude Children’s Research Hospital Committee on the Use and Care of Animals.

### Osteoclast culture

Primary osteoclasts were derived from mouse bone marrow differentiated in alpha-minimum essential medium (ThermoFisher Scientific 12571063) supplemented with 1× nonessential amino acids (ThermoFisher Scientific, 11140076), 10% FBS, and 1× penicillin–streptomycin (15070063, ThermoFisher Scientific)^74^. Bone marrow cells were flushed from the femur and tibia, and plated on plastic petri dishes with 25 ng/mL recombinant mouse macrophage colony-stimulating factor (M-CSF) for 3 days. Cells were then seeded in tissue culture plates at 1.2 × 10^4^ cells per well in 150 μL media (96-well plate) or 1.2 × 10^5^ cells per well in 1.5 mL media (12-well plate) in differentiation medium containing 25 ng/mL M-CSF and 50 ng/mL RANKL. Media was replenished after 48 h.

### Bone morphometry

Bone morphometric parameters were measured by µCT scans from age- and sex-matched mouse femurs at 6 months or 3 months of age in a blinded fashion (JK) by the Center for In Vivo Imaging and Therapeutics (CIVIT) core facility at St. Jude Children’s Research Hospital. Untreated control morphometric data was pooled from multiple independent scans, and used to compare appropriate age-matched untreated and treated mice throughout this study. µCT images were obtained using a Siemens Inveon µCT scanner (Siemens Healthcare). Mouse femurs were imaged using a 1024 × 2304 mm matrix with field of view 18.29 × 41.15 mm using one bed position. Projections were acquired at 80 kVp and 500 µA (3900 ms exposure and 3500 ms settle time) over full rotation (480 steps) providing an isotropic resolution of 17.86 µm. Data were post-processed using the segmentation tool in Inveon Research Workplace (IRW version 4.2) software to obtain morphometric measurements.

### Microscopy

Images and videos (hourly image acquisition) of osteoclasts were automatically collected using an IncuCyte S3 (EssenBiosciences). Cells were fixed in 4% PFA, permeabilized with 0.5% Triton X-100, and stained for F-actin (phalloidin-iFluor555, ab176756, Abcam, 1:2000) and nuclei (25 nM Sytox Green, S7020, ThermoFisher Scientific), according to manufacturer’s protocols. Similarly, permeabilized cells were stained for TRAP following the manufacturer’s protocol (MK301, Takara) and automatically imaged using a Nikon C2 microscope. TRAP-stained femur sections were stained by the St. Jude Children’s Research Hospital Veterinary Pathology Core and images were collected by a trained pathologist (PV).

### In vivo LPS-induced bone loss

Male mice, age-matched at 3 months, were treated with LPS (5 mg/kg) by intraperitoneal injection at days 0 and 4, and bones were collected at day 8, fixed in formalin and transferred to 70% ethanol for scanning by µCT. LPS treatment group data were compared to the pooled control age- and sex-matched scan data used throughout this study.

### Immunoblotting analysis

For signaling blots, supernatant was removed, and cells were lysed in RIPA buffer containing protease and phosphatase inhibitors plus 4× Laemmli sample buffer. Proteins were separated via SDS–PAGE with 6–12% polyacrylamide gels, transferred to PVDF membranes (IPVH00010, Millipore), and blocked with 5% nonfat dry milk. Primary antibodies against DC-STAMP (Novus Biologicals, NBP1-79329, 1:1000) or ATP6V0D2 (ThermoFischer Scientific, PA5-44359, 1:1000) were incubated overnight at 4 °C, followed by appropriate secondary antibodies conjugated with HRP (1:10000) incubated for 1 h at room temperature (Jackson ImmunoResearch, West Grove, PA). Membranes were visualized using Luminata Forte Chemiluminescence substrate (WBLUF0500, Millipore) on a BioRad ChemiDoc.

### Quantification and statistical analysis

GraphPad Prism 6.0 software was used for data analysis. Data are shown as mean ± SEM. Statistical significance was determined by Student’s *t* test for two groups or one-way analysis of variance (ANOVA) for three or more groups, and two-way ANOVA for comparison between multiple groups. The specific statistical testing for each experiment is indicated in the figure legends.

### Reporting summary

Further information on research design is available in the Nature Research Reporting Summary linked to this article.

## Supplementary information

Supplementary Information

Supplementary Movie 1

Supplementary Movie 2

Reporting Summary

Description of Additional Supplementary Files

## Data Availability

All data generated and analyzed during the current study are contained within the manuscript, and/or are available from the corresponding author on reasonable request.

## References

[1] Boyle WJ, Simonet WS, Lacey DL (2003). Osteoclast differentiation and activation. Nature.

[2] Abu-Amer Y, Ross FP, Edwards J, Teitelbaum SL (1997). Lipopolysaccharide-stimulated osteoclastogenesis is mediated by tumor necrosis factor via its P55 receptor. J. Clin. Investig..

[3] Amarasekara, D. S. et al. Regulation of osteoclast differentiation by cytokine networks. *Immune Netw*. **18**, e8 (2018). 10.4110/in.2018.18.e8 PMC5833125 29503739

[4] Bi, H. et al. Key triggers of osteoclast-related diseases and available strategies for targeted therapies: a review. *Front. Med*. **4**, 234 (2017). 10.3389/fmed.2017.00234 PMC5742334 29326938

[5] Roderick MR, Shah R, Rogers V, Finn A, Ramanan AV (2016). Chronic recurrent multifocal osteomyelitis (CRMO) – advancing the diagnosis. Pediatr. Rheumatol..

[6] Yildirim K (2010). Bone mineral density in patients with familial Mediterranean fever. Rheumatol. Int..

[7] Houx L (2015). Musculoskeletal symptoms in patients with cryopyrin-associated periodic syndromes: a large database study. Arthritis Rheumatol..

[8] Cobelli N, Scharf B, Crisi GM, Hardin J, Santambrogio L (2011). Mediators of the inflammatory response to joint replacement devices. Nat. Rev. Rheumatol..

[9] Abu-Amer Y, Darwech I, Clohisy JC (2007). Aseptic loosening of total joint replacements: mechanisms underlying osteolysis and potential therapies. Arthritis Res. Ther..

[10] How, K. Y., Song, K. P. & Chan, K. G. *Porphyromonas gingivalis*: an overview of periodontopathic pathogen below the gum line. *Front. Microbiol*. **7**, 53 (2016). 10.3389/fmicb.2016.00053 PMC4746253 26903954

[11] Kavanagh N (2018). Staphylococcal osteomyelitis: disease progression, treatment challenges, and future directions. Clin. Microbiol. Rev..

[12] Trajanoska, K. & Rivadeneira, F. The genetic architecture of osteoporosis and fracture risk. *Bone* **126**, 2–10 (2019). 10.1016/j.bone.2019.04.005 30980960

[13] Morris JA (2019). An atlas of genetic influences on osteoporosis in humans and mice. Nat. Genet..

[14] Takayanagi H (2002). RANKL maintains bone homeostasis through c-Fos-dependent induction of interferon - β. Nature.

[15] Duque G (2011). Interferon-γ plays a role in bone formation in vivo and rescues osteoporosis in ovariectomized mice. J. Bone Miner. Res..

[16] Takayanagi H, Sato K, Takaoka A, Taniguchi T (2005). Interplay between interferon and other cytokine systems in bone metabolism. Immunol. Rev..

[17] Hertzog PJ (2012). Overview. Type I interferons as primers, activators and inhibitors of innate and adaptive immune responses. Immunol. Cell Biol..

[18] Grigoriadis AE (1994). c-Fos: a key regulator of osteoclast-macrophage lineage determination and bone remodeling. Science.

[19] Gao Y (2007). IFN-gamma stimulates osteoclast formation and bone loss in vivo via antigen-driven T cell activation. J. Clin. Investig..

[20] Takayanagi H (2000). T-cell-mediated regulation of osteoclastogenesis by signalling cross-talk between RANKL and IFN-gamma. Nature.

[21] Salem S (2014). A novel role for interferon regulatory factor 1 (IRF1) in regulation of bone metabolism. J. Cell. Mol. Med..

[22] Kim S (2003). Stat1 functions as a cytoplasmic attenuator of Runx2 in the transcriptional program of osteoblast differentiation. Genes Dev..

[23] Laroche M, Livideanu C, Paul C, Cantagrel A (2011). Interferon alpha and pamidronate in osteoporosis with fracture secondary to mastocytosis. Am. J. Med..

[24] Laroche M, Bret J, Brouchet A, Mazières B (2007). Clinical and densitometric efficacy of the association of interferon alpha and pamidronate in the treatment of osteoporosis in patients with systemic mastocytosis. Clin. Rheumatol..

[25] Tang, M., Tian, L., Luo, G. & Yu, X. Interferon-gamma-mediated osteoimmunology. *Front. Immunol.* **9**, 1508 (2018). 10.3389/fimmu.2018.01508 PMC6033972 30008722

[26] Briken V (1995). Interferon regulatory factor 1 is required for mouse Gbp gene activation by gamma interferon. Mol. Cell. Biol..

[27] Huang S, Meng Q, Maminska A, MacMicking JD (2019). Cell-autonomous immunity by IFN-induced GBPs in animals and plants. Curr. Opin. Immunol..

[28] Ito Y (2010). Cdc42 regulates bone modeling and remodeling in mice by modulating RANKL/M-CSF signaling and osteoclast polarization. J. Clin. Investig..

[29] Croke M (2011). Rac deletion in osteoclasts causes severe osteopetrosis. J. Cell Sci..

[30] Lee NK, Choi HK, Kim D-K, Lee SY (2006). Rac1 GTPase regulates osteoclast differentiation through TRANCE-induced NF-kappa B activation. Mol. Cell. Biochem..

[31] Itokowa T (2011). Osteoclasts lacking Rac2 have defective chemotaxis and resorptive activity. Calcif. Tissue Int..

[32] Wang Y (2008). Identifying the relative contributions of Rac1 and Rac2 to osteoclastogenesis. J. Bone Miner. Res..

[33] Magalhaes JKRS, Grynpas MD, Willett TL, Glogauer M (2011). Deleting Rac1 improves vertebral bone quality and resistance to fracture in a murine ovariectomy model. Osteoporos. Int..

[34] Pavlos NJ (2005). Rab3D regulates a novel vesicular trafficking pathway that is required for osteoclastic bone resorption. Mol. Cell Biol..

[35] Zou W (2013). Talin1 and Rap1 are critical for osteoclast function. Mol. Cell. Biol..

[36] Russell RGG (2006). Bisphosphonates: from bench to bedside. Ann. N. Y. Acad. Sci..

[37] van Beek E, Pieterman E, Cohen L, Löwik C, Papapoulos S (1999). Farnesyl pyrophosphate synthase is the molecular target of nitrogen-containing bisphosphonates. Biochem. Biophys. Res. Commun..

[38] Li, P. et al. Ubiquitination and degradation of GBPs by a *Shigella* effector to suppress host defence. *Nature* **551**, 378–383 (2017). 10.1038/nature24467 29144452

[39] Piro, A. S. et al. Detection of cytosolic *Shigella flexneri* via a C-terminal triple-arginine motif of GBP1 inhibits actin-based motility. *m**Bio* **8**, e01979–17 (2017). 10.1128/mBio.01979-17 PMC5727416 29233899

[40] Wandel MP (2017). GBPs inhibit motility of *Shigella flexneri* but are targeted for degradation by the bacterial ubiquitin ligase IpaH9.8. Cell Host Microbe.

[41] Ostler N (2014). Gamma interferon-induced guanylate binding protein 1 is a novel actin cytoskeleton remodeling factor. Mol. Cell. Biol..

[42] Xiao L (2004). Stat1 controls postnatal bone formation by regulating fibroblast growth factor signaling in osteoblasts. J. Biol. Chem..

[43] Nishimura S (2015). MSMD patients with IFN-g-STAT1 signaling defect present enhanced osteoclastogenesis and bone resorption. Blood.

[44] Meraz MA (1996). Targeted disruption of the Stat1 gene in mice reveals unexpected physiologic specificity in the JAK-STAT signaling pathway. Cell.

[45] Bustamante J, Boisson-Dupuis S, Abel L, Casanova J-L (2014). Mendelian susceptibility to mycobacterial disease: genetic, immunological, and clinical features of inborn errors of IFN-γ immunity. Semin. Immunol..

[46] Hirata O (2013). Heterozygosity for the Y701C STAT1 mutation in a multiplex kindred with multifocal osteomyelitis. Haematologica.

[47] Decker T, Lew DJ, Cheng YS, Levy DE, Darnell JE (1989). Interactions of alpha- and gamma-interferon in the transcriptional regulation of the gene encoding a guanylate-binding protein. EMBO J..

[48] Ballanti P (1997). Tartrate-resistant acid phosphate activity as osteoclastic marker: sensitivity of cytochemical assessment and serum assay in comparison with standardized osteoclast histomorphometry. Osteoporos. Int..

[49] Zou, Z. et al. Guanylate-binding protein 1 inhibits nuclear delivery of kaposi’s sarcoma-associated herpesvirus virions by disrupting formation of actin filament. *J. Virol*. **91**, e00632-17 (2017). 10.1128/JVI.00632-17 PMC5533911 28592529

[50] Takito J, Otsuka H, Inoue S, Kawashima T, Nakamura M (2017). Symmetrical retrograde actin flow in the actin fusion structure is involved in osteoclast fusion. Biol. Open.

[51] Hurst IR, Zuo J, Jiang J, Holliday LS (2004). Actin-related protein 2/3 complex is required for actin ring formation. J. Bone Miner. Res..

[52] Place DE (2020). Interferon inducible GBPs restrict *Burkholderia thailandensis* motility induced cell-cell fusion. PLoS Pathog..

[53] Tretina K, Park E-S, Maminska A, MacMicking JD (2019). Interferon-induced guanylate-binding proteins: Guardians of host defense in health and disease. J. Exp. Med..

[54] Santos JC, Broz P (2018). Sensing of invading pathogens by GBPs: at the crossroads between cell-autonomous and innate immunity. J. Leukoc. Biol..

[55] Man SM (2015). The transcription factor IRF1 and guanylate-binding proteins target activation of the AIM2 inflammasome by *Francisella* infection. Nat. Immunol..

[56] Meunier E (2015). Guanylate-binding proteins promote activation of the AIM2 inflammasome during infection with *Francisella novicida*. Nat. Immunol..

[57] Britzen-Laurent N (2010). Intracellular trafficking of guanylate-binding proteins is regulated by heterodimerization in a hierarchical manner. PLoS ONE.

[58] Cerqueira DM (2018). Guanylate-binding protein 5 licenses caspase-11 for Gasdermin-D mediated host resistance to Brucella abortus infection. PLoS Pathog..

[59] Finethy, R. et al. Inflammasome activation by bacterial outer membrane vesicles requires guanylate binding proteins. *mBio* **8**, e01188-17 (2017). 10.1128/mBio.01188-17 PMC5626967 28974614

[60] Weivoda, M. M. & Oursler, M. J. The roles of small GTPases in osteoclast biology. *Orthop. Muscular Syst.* **3**, 1000161 (2014). 10.4172/2161-0533.1000161 PMC4296324 25599004

[61] Itzstein C, Coxon FP, Rogers MJ (2011). The regulation of osteoclast function and bone resorption by small GTPases. Small GTPases.

[62] López-Posadas, R., Stürzl, M., Atreya, I., Neurath, M. F. & Britzen-Laurent, N. Interplay of GTPases and cytoskeleton in cellular barrier defects during gut inflammation. *Front. Immunol.* **8**, 1240 (2017). 10.3389/fimmu.2017.01240 PMC5633683 29051760

[63] Lei S-F (2009). An in vivo genome wide gene expression study of circulating monocytes suggested GBP1, STAT1 and CXCL10 as novel risk genes for the differentiation of peak bone mass. Bone.

[64] Bai S, Mu Z, Huang Y, Ji P (2018). Guanylate binding protein 1 inhibits osteogenic differentiation of human mesenchymal stromal cells derived from bone marrow. Sci. Rep..

[65] Nair SP (1996). Bacterially induced bone destruction: mechanisms and misconceptions. Infect. Immun..

[66] Khor EC (2013). Loss of protein kinase C-δ protects against LPS-induced osteolysis owing to an intrinsic defect in osteoclastic bone resorption. PLOS ONE.

[67] Wu H (2018). Artemether attenuates LPS-induced inflammatory bone loss by inhibiting osteoclastogenesis and bone resorption via suppression of MAPK signaling pathway. Cell Death Dis..

[68] Meunier, E. et al. Caspase-11 activation requires lysis of pathogen-containing vacuoles by IFN-induced GTPases. *Nature* **509**, 366–370 (2014). 10.1038/nature13157 24739961

[69] Yamamoto M (2012). A cluster of interferon-γ-inducible p65 GTPases plays a critical role in host defense against Toxoplasma gondii. Immunity.

[70] Müller U (1994). Functional role of type I and type II interferons in antiviral defense. Science.

[71] Kimura T (1996). Essential and non-redundant roles of p48 (ISGF3 gamma) and IRF-1 in both type I and type II interferon responses, as revealed by gene targeting studies. Genes Cells.

[72] Matsuyama T (1993). Targeted disruption of IRF-1 or IRF-2 results in abnormal type I IFN gene induction and aberrant lymphocyte development. Cell.

[73] Durbin JE, Hackenmiller R, Simon MC, Levy DE (1996). Targeted disruption of the mouse Stat1 gene results in compromised innate immunity to viral disease. Cell.

[74] Marino, S., Logan, J. G., Mellis, D. & Capulli, M. Generation and culture of osteoclasts. *Bonekey Rep.* **3**, 570 (2014). 10.1038/bonekey.2014.65 PMC4162467 25228983

