# Supplementary material for: Osteoclast fusion and bone loss are restricted by interferon inducible guanylate binding proteins
Source: Nat Commun. 2021 Jan 21;12:496. doi: 10.1038/s41467-020-20807-8 (PMC7820603; doi:10.1038/s41467-020-20807-8)
Supplement: Supplementary file 1 — Supplementary Information [file 41467_2020_20807_MOESM1_ESM.pdf]

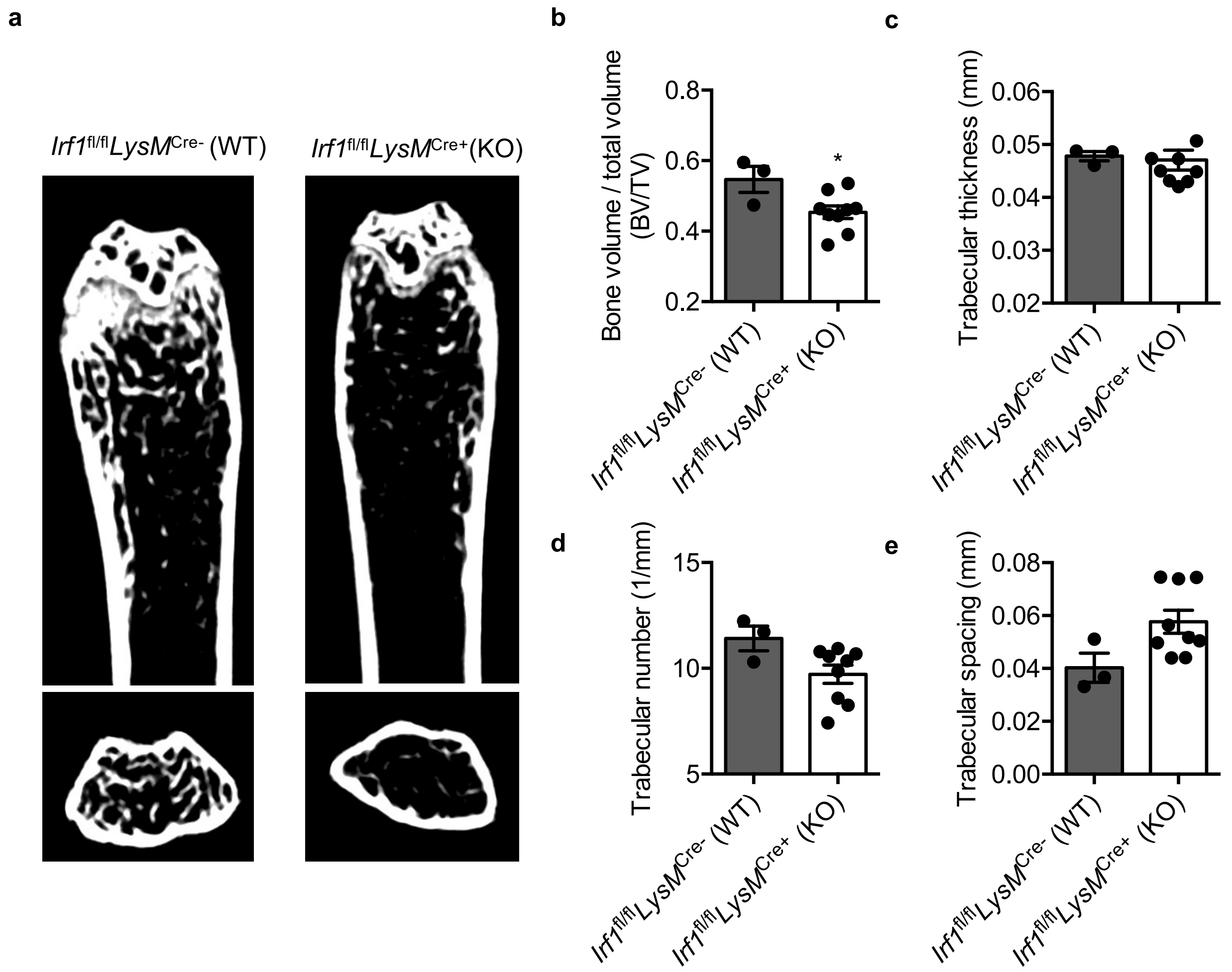

Supplementary Fig. 1. Myeloid-specific IRF1 negatively regulates bone density.

Representative 2D images (**a**) were obtained from micro-computed tomography (μCT) scans of 3-month-old littermate (**a-e**) wildtype (WT) (*Irf1*$^{fl/fl}$*LysM*$^{Cre-}$) (n=3) or myeloid-specific *Irf1*-deficient (*Irf1*$^{fl/fl}$*LysM*$^{Cre+}$) (n=9) femurs. Quantitative measures of (**b**) bone volume to total volume (BV/TV) (p=0.0332), (**c**) trabecular thickness (mm), (**d**) trabecular number (1/mm) and (**e**) trabecular spacing (mm) (p=0.0607) were obtained from independent femur scans. Statistical significance was determined by two-tailed Student's t test, *p<0.05. Data are pooled from independent scans. Data are presented as mean ± SEM (b-e).

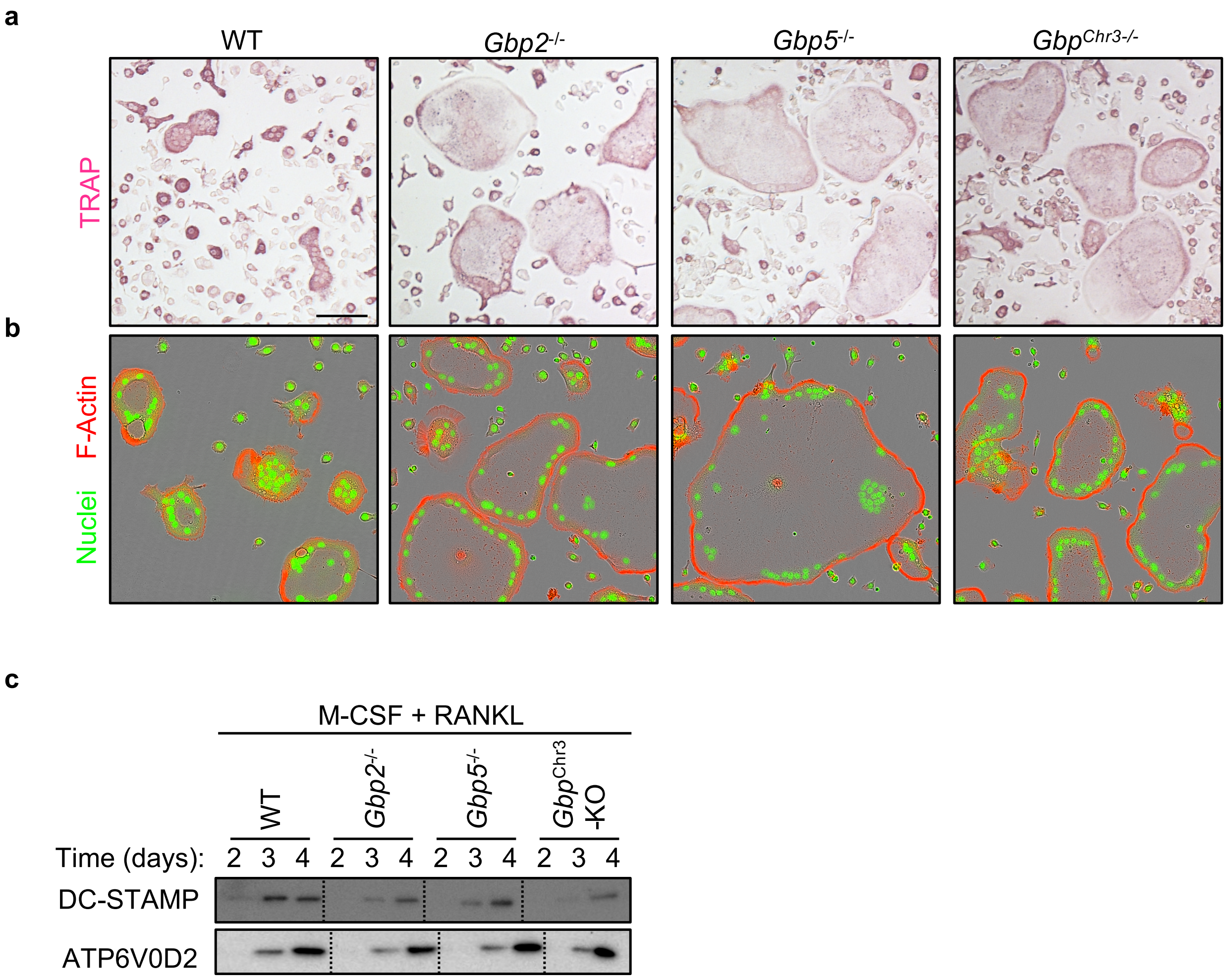

Supplementary Fig. 2. In vitro osteoclast fusion is restricted by GBP2 and GBP5. Osteoclasts were generated *in vitro* from wildtype (WT) and *Gbp2*$^{-/-}$, *Gbp5*$^{-/-}$ or *Gbp5*$^{Chr3-/-}$ bone marrow. (**a**) Osteoclasts were stained for tartrate-resistant acid phosphatase (TRAP) activity or (**b**) for F-actin (red) and nuclei (green). (**c**) Expression of essential osteoclast fusion factors (DC-STAMP and ATP6V0D2) over time was measured by immunoblot. At least nine representative images were acquired by automated microscopy using a (**a**) Nikon C2 or (**b**) IncuCyte S3. Scale bar (black) indicates 200 μm.
