# Supplementary material for: Osteoclast fusion and bone loss are restricted by interferon inducible guanylate binding proteins
Source: Nat Commun. 2021 Jan 21;12:496. doi: 10.1038/s41467-020-20807-8 (PMC7820603; doi:10.1038/s41467-020-20807-8)
Supplement: Supplementary file 4 — Reporting Summary [file 41467_2020_20807_MOESM4_ESM.pdf]

nature research

Corresponding author(s): Thirumala-Devi Kanneganti

Last updated by author(s): Oct 26, 2020

Reporting Summary

Nature Research wishes to improve the reproducibility of the work that we publish. This form provides structure for consistency and transparency in reporting. For further information on Nature Research policies, see our Editorial Policies and the Editorial Policy Checklist.

Statistics

For all statistical analyses, confirm that the following items are present in the figure legend, table legend, main text, or Methods section.

n/a	Confirmed	
☐	☒	The exact sample size (n) for each experimental group/condition, given as a discrete number and unit of measurement
☐	☒	A statement on whether measurements were taken from distinct samples or whether the same sample was measured repeatedly
☐	☒	The statistical test(s) used AND whether they are one- or two-sided<br>*Only common tests should be described solely by name; describe more complex techniques in the Methods section.*
☐	☒	A description of all covariates tested
☐	☒	A description of any assumptions or corrections, such as tests of normality and adjustment for multiple comparisons
☐	☒	A full description of the statistical parameters including central tendency (e.g. means) or other basic estimates (e.g. regression coefficient) AND variation (e.g. standard deviation) or associated estimates of uncertainty (e.g. confidence intervals)
☐	☒	For null hypothesis testing, the test statistic (e.g. F, t, r) with confidence intervals, effect sizes, degrees of freedom and P value noted<br>*Give P values as exact values whenever suitable.*
☒	☐	For Bayesian analysis, information on the choice of priors and Markov chain Monte Carlo settings
☐	☒	For hierarchical and complex designs, identification of the appropriate level for tests and full reporting of outcomes
☒	☐	Estimates of effect sizes (e.g. Cohen's d, Pearson's r), indicating how they were calculated

Our web collection on statistics for biologists contains articles on many of the points above.

Software and code

Policy information about availability of computer code

Data collection	Bone morphometric parameters were measured by µCT scans from age- and sex-matched mouse femurs at 6 months or 3 months of age in a blinded fashion (by JK) by the Center for In Vivo Imaging and Therapeutics (CIVIT) core facility at St. Jude Children's Research Hospital. Untreated control morphometric data were pooled from multiple independent scans and used to compare appropriate age-matched untreated and treated mice throughout this study. µCT images were obtained using a Siemens Inveon µCT scanner (Siemens Healthcare). Mouse femurs were imaged using a 1024 x 2304 mm matrix with Field of View (FOV) 18.29 x 41.15 mm using 1 bed position. Projections were acquired at 80 kVp and 500 µA (3900 ms exposure and 3500 ms settle time) over full rotation (480 steps) providing an isotropic resolution of 17.86 µm. Data were post-processed using the segmentation tool in Inveon Research Workplace (IRW version 4.2) software to obtain morphometric measurements.<br><br>Images and videos (hourly image acquisition) of osteoclasts were automatically collected using an IncuCyte S3 (EssenBiosciences). Cells were fixed in 4% PFA, permeabilized with 0.5% Triton X-100 and stained for F-actin (phalloidin-iFluor555, ab176756, Abcam, 1:2000) and nuclei (25 nM Sytox Green, S7020, ThermoFisher Scientific) according to manufacturer's protocols. Similarly, permeabilized cells were stained for TRAP following the manufacturer's protocol (MK301, Takara) and automatically imaged using a Nikon C2 microscope. TRAP-stained femur sections were stained by the St. Jude Children's Research Hospital Veterinary Pathology Core and images were collected by a trained pathologist (PV).<br><br>GraphPad Prism 6.0 software was used for data analysis. Data are shown as mean ± SEM. Statistical significance was determined by Student's t test for two groups or one-way analysis of variance (ANOVA) for three or more groups and two-way ANOVA for comparison between multiple groups. The specific statistical testing for each experiment is indicated in the figure legends.
Data analysis	Bone morphometric parameters were measured by µCT scans from age- and sex-matched mouse femurs at 6 months or 3 months of age in a blinded fashion (by JK) by the Center for In Vivo Imaging and Therapeutics (CIVIT) core facility at St. Jude Children's Research Hospital. Untreated control morphometric data were pooled from multiple independent scans and used to compare appropriate age-matched untreated and treated mice throughout this study. µCT images were obtained using a Siemens Inveon µCT scanner (Siemens Healthcare).

Mouse femurs were imaged using a 1024 x 2304 mm matrix with Field of View (FOV) 18.29 x 41.15 mm using 1 bed position. Projections were acquired at 80 kVp and 500 µA (3900 ms exposure and 3500 ms settle time) over full rotation (480 steps) providing an isotropic resolution of 17.86 µm. Data were post-processed using the segmentation tool in Inveon Research Workplace (IRW version 4.2) software to obtain morphometric measurements.

Images and videos (hourly image acquisition) of osteoclasts were automatically collected using an IncuCyte S3 (EssenBiosciences). Cells were fixed in 4% PFA, permeabilized with 0.5% Triton X-100 and stained for F-actin (phalloidin-iFluor555, ab176756, Abcam, 1:2000) and nuclei (25 nM Sytox Green, S7020, ThermoFisher Scientific) according to manufacturer's protocols. Similarly, permeabilized cells were stained for TRAP following the manufacturer's protocol (MK301, Takara) and automatically imaged using a Nikon C2 microscope. TRAP-stained femur sections were stained by the St. Jude Children's Research Hospital Veterinary Pathology Core and images were collected by a trained pathologist (PV).

GraphPad Prism 6.0 software was used for data analysis. Data are shown as mean ± SEM. Statistical significance was determined by Student's t test for two groups or one-way analysis of variance (ANOVA) for three or more groups and two-way ANOVA for comparison between multiple groups. The specific statistical testing for each experiment is indicated in the figure legends.

For manuscripts utilizing custom algorithms or software that are central to the research but not yet described in published literature, software must be made available to editors and reviewers. We strongly encourage code deposition in a community repository (e.g. GitHub). See the Nature Research guidelines for submitting code & software for further information.

Data

Policy information about availability of data

All manuscripts must include a data availability statement. This statement should provide the following information, where applicable:

- Accession codes, unique identifiers, or web links for publicly available datasets
- A list of figures that have associated raw data
- A description of any restrictions on data availability

All data generated and analyzed during the current study are contained within the manuscript and/or are available from the corresponding author on reasonable request.

Field-specific reporting

Please select the one below that is the best fit for your research. If you are not sure, read the appropriate sections before making your selection.

☒ Life sciences ☐ Behavioural & social sciences ☐ Ecological, evolutionary & environmental sciences

For a reference copy of the document with all sections, see nature.com/documents/nr-reporting-summary-flat.pdf

Life sciences study design

All studies must disclose on these points even when the disclosure is negative.

Sample size	Sample size was not pre-determined before experiments. In vitro experiments were performed with technical replicates and independent biological replicates. If replicate experiments resulted in similar results, experiments were considered reproducible.
Data exclusions	No data points were excluded from analysis. No data were excluded except in the case of bone fracture during handling of bone samples for uCT.
Replication	Experimental data were replicated in vitro as indicated and micro-CT data were pooled, where appropriate, from data collected from multiple independent groups of mice that were age- and sex-matched. Micro-CT data were pooled from multiple independent replicate groups of mice. All experimental replications were successful. Data were largely consistent with previous publications from other research groups where similar knockout models were examined, and contrasting findings were discussed in the manuscript text.
Randomization	Experimental samples were not randomized; however, age- and sex-matched femur samples were collected from mice with appropriate age, sex, and genotype as they became available. Within samples, individual images used for quantification were collected randomly (Nikon C2) or in an automated fashion according to a standard imaging pattern per well (via Incucyte).
Blinding	Micro-CT scan morphometry data were collected and analysed by a blinded technician and unblinded to perform statistical analysis and assign genotypes to samples. Bone histology images were collected by a blinded veterinary pathologist and unblinded to assign genotypes to samples. All other data experiments were performed unblinded to assure proper genotypes were used and age- and sex-matching was performed.

Reporting for specific materials, systems and methods

We require information from authors about some types of materials, experimental systems and methods used in many studies. Here, indicate whether each material, system or method listed is relevant to your study. If you are not sure if a list item applies to your research, read the appropriate section before selecting a response.

Materials & experimental systems

n/a	Involved in the study	
☐	☒	Antibodies
☒	☐	Eukaryotic cell lines
☒	☐	Palaeontology and archaeology
☐	☒	Animals and other organisms
☒	☐	Human research participants
☒	☐	Clinical data
☒	☐	Dual use research of concern

Methods

n/a	Involved in the study	
☒	☐	ChIP-seq
☒	☐	Flow cytometry
☒	☐	MRI-based neuroimaging

Antibodies

Antibodies used

Immunoblotting Analysis
Primary antibodies against DC-STAMP (Novus Biologicals, NBP1-79329, 1:1000) or ATP6V0D2 (ThermoFischer Scientific, PA5-44359, 1:1000) were used. Secondary anti-rabbit (111-03-047 from Jackson ImmunoResearch Laboratories; 1:10000) was used.

Microscopy (non-antibody fluorescent probe)
Phalloidin-iFluor 555 reagent (Abcam, ab176756, 1:2000)

Validation

ATP6V0D2: Per manufacturer website, 293T cells were transfected and western blot analysis was performed with antibody.
DC-STAMP: Per manufacturer website, DC-STAMP antibody was validated using OVCAR-3 cell lysate by western blot.
Phalloidin-iFluor 555 reagent: Per manufacturer website, phalloidin-iFluor 555 reagent was validated by staining HeLa cells, independently validated in extensive publications, our previous studies, and is a well-established method for staining F-actin.

Animals and other organisms

Policy information about studies involving animals; ARRIVE guidelines recommended for reporting animal research

Laboratory animals

Wildtype (C57BL/6J), Gbp2−/− (Gbp2tm1b(KOMP)Wtsi), Gbp5−/−, GbpChr3−/− (MGI:5438974)68, Ifnar1−/− (Ifnar1tm1Agt)69, Irf9−/− (Irf9tm1Ttg)70, Irf1−/− (Irf1tm1Mak)71, Stat1−/− (Stat1tm1Dlv)72 mice backcrossed to the B6 background have been described previously55,73. Myeloid-specific Irf1 knockout mice (Irf1fl/flLysMCre+) were generated by crossing Irf1fl/fl mice (B6Dnk;B6Brd;B6N-Tyrc-BrdIrf1tm1a(EUCOMM)Wtsi/WtsiOulu; Infrafrontier, EM:05519) and LysMCre+ mice (B6.129P2-Lyz2tm1(cre)Ifo/J), and littermate controls were used. Male mice were used in this study at indicated ages (3 or 6 months) or 6-8 weeks old (for generation of in vitro osteoclasts). Mouse colonies were maintained at 21°C and 60%–64% humidity, on a 12 hour lights on/off cycle. Mice were bred at St. Jude Children's Research Hospital, and studies were conducted under protocols approved by St. Jude Children's Research Hospital on the Use and Care of Animals.

Wild animals

This study did not involve wild animals.

Field-collected samples

This study did not involve field-collected samples.

Ethics oversight

Studies were conducted under protocols approved by St. Jude Children's Research Hospital Institutional Committee on the Use and Care of Animals, protocol number 482. It is the policy of the St. Jude Children's Research Hospital Animal Care and Use Committee that all research involving animals be conducted according to the highest possible professional, ethical, and scientific standards and that all animals be housed, maintained, and handled in compliance with the standards set forth by the Animal Welfare Act of 1966 (9 CFR Part 3 as amended); the National Research Council 1996 "Guide for the Care and Use of Laboratory Animals"; the Public Health Service Policy on the Humane Care and Use of Laboratory Animals (revised September 1986); the United States Government Principles for the Utilization and Care of Vertebrate Animals Used in Testing, Research, and Training; the report of the American College of Laboratory Animal Medicine on Adequate Veterinary Care in Research, Testing, and Teaching; the 2007 Guidelines on Euthanasia; and all other applicable federal, state, and local laws, regulations, and policies. St. Jude Children's Research Hospital is committed to maintaining full accreditation status by AAALAC International.

Note that full information on the approval of the study protocol must also be provided in the manuscript.
