# Supplementary material for: Osteoclast fusion and bone loss are restricted by interferon inducible guanylate binding proteins
Source: Nat Commun. 2021 Jan 21;12:496. doi: 10.1038/s41467-020-20807-8 (PMC7820603; doi:10.1038/s41467-020-20807-8)
Supplement: Supplementary file 5 — Description of Additional Supplementary Files [file 41467_2020_20807_MOESM5_ESM.pdf]

Title: Supplementary Movie 1. WT osteoclast differentiation

Wildtype (WT) osteoclasts were differentiated *in vitro.* After 48 h stimulation with M-CSF and RANKL, cells were imaged hourly on an IncuCyte S3 at 20x magnification to visualize osteoclast fusion.

Title: Supplementary Movie 2. *Gbp*$^{\mathrm{Chr3-/-}}$ osteoclast differentiation

Gbp$^{\mathrm{Chr3-/-}}$ osteoclasts were differentiated *in vitro.* After 48 h stimulation with M-CSF and RANKL, cells were imaged hourly on an IncuCyte S3 at 20x magnification to visualize osteoclast fusion.
